# Benralizumab: From the Basic Mechanism of Action to the Potential Use in the Biological Therapy of Severe Eosinophilic Asthma

**DOI:** 10.1155/2018/4839230

**Published:** 2018-05-10

**Authors:** Corrado Pelaia, Cecilia Calabrese, Alessandro Vatrella, Maria Teresa Busceti, Eugenio Garofalo, Nicola Lombardo, Rosa Terracciano, Girolamo Pelaia

**Affiliations:** ^1^Dipartimento di Scienze Mediche e Chirurgiche, Università degli Studi “Magna Græcia”, Catanzaro, Italy; ^2^Dipartimento di Scienze Cardio-Toraciche e Respiratorie, Università degli Studi della Campania “Luigi Vanvitelli”, Naples, Italy; ^3^Dipartimento di Medicina, Chirurgia ed Odontoiatria, Università degli Studi di Salerno, Salerno, Italy; ^4^Dipartimento di Scienze della Salute, Università degli Studi “Magna Græcia”, Catanzaro, Italy

## Abstract

Asthma is a very frequent chronic airway disease that includes many different clinical phenotypes and inflammatory patterns. In particular, eosinophilic bronchial inflammation is often associated with allergic as well as nonallergic asthma. The most important cytokine involved in the induction, maintenance, and amplification of airway eosinophilia in asthma is interleukin-5 (IL-5), released by both T helper 2 (Th2) lymphocytes and group 2 innate lymphoid cells (ILC2). Hence, IL-5 and its receptor are suitable targets for selective biologic drugs which can play a key role in add-on treatment of severe eosinophilic asthma refractory to corticosteroids. Within such a context, the anti-IL-5 monoclonal antibodies mepolizumab and reslizumab have been developed and approved for biological therapy of uncontrolled eosinophilic asthma. In this regard, on the basis of several successful randomized controlled trials, the anti-IL-5 receptor benralizumab has also recently obtained the approval from US Food and Drug Administration (FDA).

## 1. Introduction

Asthma is a chronic obstructive airway disorder characterized by inflammatory and structural changes which cause a usually reversible airflow limitation responsible for recurrent episodes of dyspnea, wheezing, and chest tightness [1–4]. This very common disease affects more than 300 million people worldwide and originates from complex interactions between genetic and environmental factors [2]. The resulting phenotypes/endotypes include different patterns of airway inflammation, among which the eosinophilic subtype is quite frequent. Indeed, although the exact prevalence of eosinophilic asthma is not known, among patients with severe asthma who represent about 5–10% of the entire asthmatic population, eosinophilia in either sputum (≥2%) or blood (≥300 cells/*μ*l) can be detected within a 32–40% range [5, 6]. In many asthmatic patients, airway eosinophilia develops as a consequence of the biological activity of both Th2 and ILC2 cells, which are crucially implicated in the pathogenesis of type-2 inflammation underlying eosinophilic allergic and nonallergic asthma. Differently from neutrophilic asthma mainly sustained by non-type-2 mechanisms driven by Th1 and especially Th17 cells, type-2 eosinophilic asthma is generally well controlled by corticosteroids that induce the apoptotic death of eosinophils via inhibition of the production of essential survival cytokines for these cells, such as IL-5, IL-3, and granulocyte macrophage colony-stimulating factor (GM-CSF) [7, 8]. Nevertheless, some patients with eosinophilic airway inflammation are refractory to corticosteroids, thus manifesting a severe, uncontrolled asthma featured by recurrent exacerbations. It is very likely that in these subjects with difficult-to-treat eosinophilic asthma a very intense activation of type-2 inflammatory pathways occurs, leading to an exaggerate overexpression of IL-5, which remarkably decreases eosinophil sensitivity to corticosteroids [9]. In fact, the proapoptotic effect exerted by corticosteroids on eosinophils vanishes when these cells are subjected to the antiapoptotic action of high IL-5 levels [10].

These considerations thus strongly indicate that IL-5 is of paramount importance for the development, persistence, and amplification of eosinophilic asthma associated with a type-2 inflammatory/immune response. This implies that IL-5 and its receptor currently represent key molecules to be targeted by useful biological drugs for the treatment of severe eosinophilic asthma [11, 12]. Indeed, two humanized anti-IL-5 monoclonal antibodies (i.e., mepolizumab and reslizumab) have been designed, developed, and approved for add-on therapy of refractory eosinophilic asthma, whereas a third biologic drug (benralizumab) acts via a blockade of the IL-5 receptor [13–20]. These pharmacological options may thereby satisfy the unmet needs of patients with severe eosinophilic asthma who cannot achieve an adequate control of their disease, despite the use of high doses of therapeutic combinations of inhaled corticosteroids (ICS) and long-acting *β*_2_-adrenergic agonists (LABA), eventually integrated by the addition of other controller medications including inhaled anticholinergics, as well as oral corticosteroids and leukotriene inhibitors [21, 22].

Therefore, the aim of the present review article is to briefly outline the role of IL-5 in eosinophilic asthma and especially to discuss the mechanism of action and the clinical effects of the IL-5 receptor antagonist benralizumab.

## 2. The Pathobiologic Role of IL-5 in Eosinophilic Asthma

IL-5 is mainly produced by Th2 lymphocytes and ILC2 cells [27–31]. Th2 lymphocytes synthesize and release IL-5 when they are activated by antigen-presenting dendritic cells, in the presence of IL-4 acting via stimulation of the transcription factors STAT6 and GATA3, whilst ILC2 produce IL-5 upon activation mediated by airway epithelium-derived alarmins including IL-25, IL-33, and thymic stromal lymphopoietin (TSLP), which also activate GATA3 [32].

**Figure 1:**
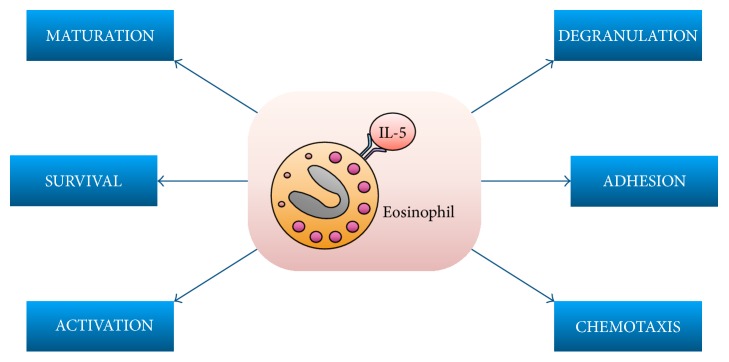
Main biological effects exerted by IL-5 on eosinophils.

The main biological effects exerted by IL-5 on eosinophils are summarized in Figure 1. In the bone marrow, as well as in the airways of patients with allergic asthma, IL-5 induces eosinophil differentiation and maturation from CD34+ hematopoietic progenitor cells [33, 34]. Indeed, high amounts of IL-5, eosinophil progenitors, and mature eosinophils can be found in the induced sputum obtained from these subjects. Moreover, by synergistically cooperating with powerful chemoattractants for eosinophils such as eotaxins 1, 2, and 3, IL-5 significantly contributes to the recruitment of these cells into the airways of asthmatic patients [15]. In particular, IL-5 promotes the interaction of eosinophils with vascular endothelial cells and their subsequent extravasation by upregulating the expression of key adhesion molecules such as *α*_L_ integrin (CD11a), *α*_M_ integrin (CD11b), *β*_2_ integrin (CD18), and P-selectin glycoprotein ligand-1 (PSGL-1/CD162) [35]. Recruitment of eosinophils into asthmatic airways is also favoured by IL-5-induced eosinophil adhesion to protein components of the extracellular matrix like periostin, whose expression is stimulated by type-2 inflammatory/immune response [35]. When released from ILC2 cells, IL-5 is also remarkably involved in the pathogenesis of late onset, nonallergic eosinophilic asthma [29, 30].

The biological actions performed by IL-5 on eosinophils are mediated by activation of the IL-5 receptor, a membrane protein consisting of two components, an *α* subunit (IL-5R*α*), which is specific for IL-5, and a *β*c chain, which can bind not only IL-5 but also IL-3 and GM-CSF [36–40]. IL-5 binding to IL-5R*α* induces the dimerization of *α* and *β*c receptor components and the subsequent activation of a complex signaling network including Janus kinase 2 (JAK2) with the associated signal transducers and activators of transcription (STAT) 1, 3, and 5, as well as several other kinases such as Lyn and Raf-1, mitogen-activated protein kinases (MAPK), phosphoinositide 3-kinase (PI3K), and protein kinase C (PKC) [41–50]. Through these mechanisms, IL-5 stimulates eosinophil differentiation, survival, proliferation, adhesion, chemoattraction, and degranulation, the latter being responsible for the release of cytotoxic proteins such as eosinophil cationic protein (ECP), major basic protein (MBP), eosinophil-derived neurotoxin (EDN), and eosinophil peroxidase (EP), which damage the airway epithelial cell layer. As a result of these cellular processes, high eosinophil counts can be found in bronchial specimens, induced sputum, and peripheral blood of patients with asthma triggered by type-2 inflammatory pathways [51–54].

## 3. Benralizumab: Mechanism of Action

**Figure 2:**
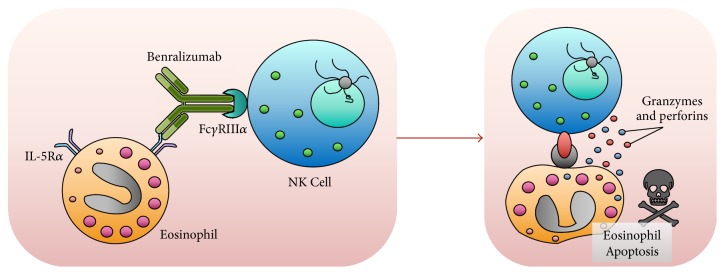
Mechanisms of action of benralizumab. Via its Fab fragments, the humanized monoclonal antibody benralizumab specifically binds to IL-5R*α*, thereby preventing the interaction between IL-5 and its receptor. In addition, through its Fc constant region, benralizumab binds to the Fc*γ*IIIRa receptor expressed by natural killer cells, thus inducing eosinophil apoptosis operated by the release of proapoptotic proteins such as granzymes and perforins.

Benralizumab (MEDI-563) was designed and produced by AstraZeneca/MedImmune by means of hybridoma technology [55–57]. This biologic drug is a humanized IgG1k monoclonal antibody generated in mouse and characterized by the specific feature of selectively binding to the amino acid residue isoleucine-61 included in domain 1 of human IL-5R*α*. Via this linkage, benralizumab thus interacts with an extracellular IL-5R*α* epitope that is situated in close proximity of the IL-5 binding site [58, 59]. This high-affinity interaction between benralizumab and IL-5R*α* impedes IL-5 binding to its receptor and the consequent heterodimerization of *α* and *β*c subunits (Figure 2), thereby preventing the stimulation of the intricate signaling mechanisms coupled to IL-5 receptor activation.

Besides linking IL-5R*α* through its Fab regions, benralizumab also binds via the constant Fc fragment to the Fc*γ*RIIIa receptor, located on cell membrane of natural killer (NK) cells (Figure 2) [60, 61]. In this regard, it is worth noticing that benralizumab was developed in Chinese hamster ovary cells not expressing the *α*-1,6-fucosyltransferase enzyme. As a consequence, lack of the fucose molecule in the sugar component of the CH2 domain of the constant segment of the monoclonal antibody is responsible for a remarkable enhancement (5 to 50 times) of benralizumab affinity for the Fc*γ*RIIIa receptor of NK cells [59]. In particular, with regard to the original fucosylated antibody, afucosylation makes benralizumab capable of inducing a ≥1000-fold amplification of the apoptotic mechanism named antibody-dependent cell-mediated cytotoxicity (ADCC). Indeed, benralizumab is a potent inducer of eosinophil apoptosis operated by NK cells through the release of the proapoptotic proteins perforin and granzyme B (Figure 2) [59]. Afucosylation-dependent ADCC has also been demonstrated by benralizumab-induced increase in eosinophil staining with annexin V, a well-known biomarker of apoptosis [59].

It can thus be argued that benralizumab is capable of killing eosinophils via a dual mechanism: the blockade of IL-5-mediated survival of these cells and the enhancement of eosinophil apoptosis induced by activation of the Fc*γ*RIIIa receptor of NK cells (Figure 2). By acting via such very powerful modalities, benralizumab rapidly and effectively depletes eosinophils in patients with asthma, thereby drastically reducing cell counts in both airways and peripheral blood [27].

## 4. Benralizumab: Clinical Trials in Asthma

In an early phase 1 study, Busse et al. evaluated the pharmacokinetics and pharmacodynamics of benralizumab by measuring eosinophil counts in peripheral blood throughout 12 weeks after intravenous injection to subjects with mild asthma of single doses ranging from 0.0003 to 3 mg/kg [62]. Benralizumab induced a dose-dependent and long-lasting depletion of blood eosinophils, which persisted at least 8 or 12 weeks at dosages of 0.03 to 0.1 or 0.3 to 3 mg/kg, respectively, whilst no significant effect was elicited by doses included within a range of 0.0003–0.003 mg/kg. Utilized at dosages ranging from 0.03 mg/kg to 3 mg/kg, the mean maximum concentration (1–82 *μ*g/mL) of benralizumab resulted to be dose-proportional. The mean distribution volume (52–93 mL/kg) of the drug was greater than the plasma volume, thus suggesting that benralizumab probably binds to circulating cells that express IL-5R*α*, and can also moderately penetrate into extravascular tissues. Consistent with the pharmacokinetic profile of human IgG antibodies, benralizumab had a mean elimination half-life of 2-3 weeks.

Another phase 1 trial was later carried out by Laviolette et al. who studied 26 asthmatics enrolled because of their ascertained airway hyperresponsiveness or reversible airflow limitation [63]. 8 participants, treated with a single intravenous injection of 1 mg/kg of benralizumab, were comparatively evaluated with 5 patients randomized to receive placebo. Moreover, benralizumab was subcutaneously administered every month for 3 months at dosages of 100 and 200 mg to 4 and 5 subjects, respectively, who were compared to 4 patients treated with placebo. In order to assess airway eosinophilia, bronchial mucosal and submucosal samples were obtained via bronchoscopy at baseline and 28 days after the conclusion of therapy. In addition, eosinophil counts were also performed in peripheral blood. In some patients, eosinophils were also measured in induced sputum and bone marrow specimens. Given either intravenously or subcutaneously, benralizumab effectively depleted eosinophils in both peripheral blood and bone marrow. When compared to placebo, benralizumab also lowered eosinophil counts in induced sputum and bronchial samples, though such reductions did not result to be statistically significant. Hence, these findings indicate that, differently from bone marrow cells, which were completely sensitive to benralizumab, bronchial eosinophils did not completely respond to this drug. Therefore, it could be inferred that airway eosinophils, because of their more advanced stage throughout the maturation process, are less responsive to biological therapies targeting either IL-5 or its receptor [64]. Benralizumab was also capable of significantly decreasing the numbers of IL-5 receptor expressing blood basophils [63], which have been shown to be increased in bronchial walls of allergic asthmatic patients [65, 66].

Subsequently, a phase 2a study was conducted by Park et al. in asthmatic subjects with ≥ 2% sputum eosinophils or FeNO ≥ 50 ppb (parts per billion), treated with medium/high doses of ICS/LABA combinations, who had manifested 2–6 exacerbations of asthma during the previous 12 months [67]. In particular, 27 patients were randomly assigned to receive placebo, whereas benralizumab was administered via the subcutaneous route at doses of 2 mg, 20 mg, and 100 mg to 27, 26, and 26 subjects, respectively. Treatments with either drug or placebo were carried out at baseline and after 4, 8, 16, 24, 32, and 40 weeks. When compared to placebo, at the 52nd week, benralizumab decreased the annual asthma exacerbation rates by 33, 45, or 36% when utilized at dosages of 2, 20, or 100 mg, respectively. Moreover, lung function was improved during this study by all dosages of benralizumab, which enhanced forced expiratory volume in one second (FEV_1_), thus inducing the highest increase at the 52nd week after administration of the 100 mg dosage, especially in patients having blood eosinophil levels ≥ 300 cells/*μ*L (mean FEV_1_ increase: 28.1%). Furthermore, benralizumab markedly reduced blood eosinophil counts. At all dosages, benralizumab was well tolerated and its safety profile did not result to be significantly different with respect to placebo.

Castro et al. performed a phase 2b study, which was completed by 324 eosinophilic and 282 noneosinophilic patients with uncontrolled asthma, treated with medium-high doses of ICS/LABA combinations, who during the previous 12 months had manifested 2–6 disease exacerbations [68]. Eosinophilic patients were randomly subdivided into four groups, assigned to receive placebo (*n* = 80) or benralizumab at dosages of 2 (*n* = 81), 20 (*n* = 81), or 100 mg (*n* = 82), respectively. Noneosinophilic subjects were randomized to receive either placebo (*n* = 142) or benralizumab (*n* = 140) at a dose of 100 mg, respectively. Both benralizumab and placebo were injected subcutaneously every 4 weeks with regard to the first 3 administrations (1st, 4th, and 8th weeks) and subsequently every 8 weeks (16th, 24th, 32nd, and 40th weeks). When compared with placebo, at the 52nd week, benralizumab decreased the annual rates of asthma exacerbations in eosinophilic participants who had been treated with drug dosages of 100 mg but not 20 mg or 2 mg. Benralizumab lowered asthma exacerbations to a greater extent in patients with blood eosinophil numbers ≥ 300 cells/*μ*L. In these subjects, exacerbations were decreased by both drug doses of 100 mg and 20 mg. In noneosinophilic asthmatics, at the dosage of 100 mg, benralizumab did not reduce the annual exacerbation rate. In eosinophilic patients, all dosages of benralizumab lowered blood eosinophil numbers, improved symptom control, and enhanced FEV_1_ values. With respect to placebo; benralizumab caused a slightly higher number of mild-to-moderate adverse reactions; nasopharyngitis and local irritations at injection sites were the most frequently observed events.

Another phase 2 study was performed by Nowak et al. who assessed the impact on hospitalization for acute asthma and/or on recurrence of disease exacerbations of a single intravenous administration of benralizumab prescribed as add-on biological therapy on discharge from emergency department [69]. The 108 participants who completed the study were subdivided into 3 groups of 36 patients, randomly assigned to receive either placebo or benralizumab at a dosage of 0.3 mg/kg or 1.0 mg/kg, respectively. The recruited asthmatic subjects were selected on the basis of specific features referring to the previous year, including at least one asthma exacerbation needing an urgent care visit, as well as an access to emergency department for acute asthma, only partially responsive to standard therapy. When compared with placebo, after 12 weeks of treatment, benralizumab induced significant decreases of 49% and 60% in the rates of asthma exacerbations and exacerbations requiring hospitalization, respectively. These effects were associated with drastic reductions in blood eosinophil counts and also in serum concentrations of the eosinophilic cytotoxic proteins ECP and EDN. Such findings were reported with regard to administration of both benralizumab dosages (0.3 mg/kg and 1.0 mg/kg). Benralizumab was characterized by a good safety pattern. In fact, only mild-to-moderate and self-limiting adverse reactions occurred, such as cough, bronchitis, fever, headache, muscle spasms, dizziness, hyperhidrosis, and anxiety. After 12 weeks of treatment, anti-benralizumab antibodies were found in 6 patients, but no clinical consequence was reported. In addition to Nowak et al., also Pham et al. showed that benralizumab significantly reduced the serum concentrations of ECP and EDN [70]. Therefore, such results further validate the concept that benralizumab can be able not only to decrease blood eosinophil counts but also to inhibit eosinophil degranulation and the consequent release of cytotoxic proteins.

In addition to phase 1 and phase 2 trials, many phase 3 studies have recently led to the approval of benralizumab by US FDA for the add-on biological therapy of severe eosinophilic asthma. In this regard, six phase 3 trials (SIROCCO, CALIMA, ZONDA, BORA, BISE, and GREGALE) are included within the so-called WINDWARD program [19]. The main phase 3 trials are summarized in Table 1.

In the SIROCCO study, Bleecker et al. randomized 1205 asthmatics, receiving high doses of ICS/LABA, to be treated for 48 weeks with one of the following add-on subcutaneous therapies: 407 subjects received placebo; 400 participants were treated with 30 mg of benralizumab every four weeks (Q4W); 398 patients were assigned to receive 30 mg of benralizumab every eight weeks (Q8W) [23]. When compared with placebo, after 48 weeks of treatment, benralizumab lowered the annual rates of asthma exacerbations by 45% and 51% in participants belonging to Q4W and Q8W groups with ≥300 blood eosinophils/*μ*L, respectively. It is also worth noticing that the annual rate of asthma exacerbations diminished by 17–30% in patients with <300 blood eosinophils/*μ*L. Furthermore, with respect to placebo, at the 48th week, both regimens of benralizumab significantly increased prebronchodilator FEV_1_, with mean changes above baseline values of 106 mL and 159 mL in Q4W and Q8W dosage schemes, respectively. When compared to placebo, benralizumab induced a better control of asthma symptoms only in the Q8W group. The most commonly reported adverse reactions were nasopharyngitis detected in 12% of subjects receiving either placebo or benralizumab and worsening of asthma observed in 13% of patients receiving benralizumab and in 19% of participants treated with placebo, respectively.

FitzGerald et al. carried out the CALIMA trial, another study that recruited patients with asthma not adequately controlled by medium-to-high doses of ICS/LABA combinations who manifested two or more disease exacerbations during the previous 12 months [24]. Similar to the SIROCCO study, throughout 56 weeks, 440 participants received placebo, whilst 425 and 441 patients were treated subcutaneously with 30 mg of benralizumab every four (Q4W) or eight weeks (Q8W), respectively. When compared with placebo, Q4W and Q8W dosage schemes significantly lowered the annual rates of asthma exacerbations by 36% and 28%, respectively. Furthermore, in subjects having ≥300 blood eosinophils/*μ*L, Q4W and Q8W regimens induced mean improvements in prebronchodilator FEV_1_ of 125 mL and 116 mL, respectively. Moreover, both dosages of benralizumab drastically decreased blood eosinophil numbers, whereas only the Q8W schedule elicited a better control of asthma symptoms with respect to placebo. Nasopharyngitis (21% in the Q4W regimen, 18% in the Q8W arm, and 21% in the placebo group, resp.) and asthma worsening (14% in the Q4W cohort, 11% in the Q8W regimen, and 15% in the placebo group, resp.) were the most frequent adverse events.

Pooled results from SIROCCO and CALIMA trials have been recently analyzed by Chipps et al. who showed that benralizumab effectively decreased asthma exacerbations and also improved both lung function and quality of life in patients with eosinophilic asthma, regardless of serum IgE levels and atopic status [71]. Therefore, these findings indicate that benralizumab can be very useful for the management of severe eosinophilic asthma, associated or not associated with an allergic trait.

Nair et al. conducted the ZONDA study with the aim of evaluating, in severe uncontrolled asthmatics, the eventual ability of benralizumab to decrease the consumption of oral corticosteroids [25]. In particular, 220 patients were randomly assigned to a subcutaneous treatment, performed every 4 (Q4W) or 8 (Q8W) weeks for 28 weeks, with either placebo or benralizumab 30 mg. At the beginning of the trial, all participants were on maintenance treatment with oral glucocorticoids (median dose: 10 mg/day; range: 7.5–40 mg/day), whose median daily dosage resulted to be reduced at the end of the study by 75% in both groups of patients receiving benralizumab and by 25% in subjects treated with placebo, respectively (*p* < 0.001) [25]. Moreover, with respect to placebo, benralizumab lowered the annual rates of asthma exacerbations by 55% and 70% in Q4W and Q8W subgroups, respectively. Benralizumab and placebo did not differ with regard to their effects on FEV_1_. Finally, benralizumab and placebo were characterized by similar profiles of safety and tolerability; with both treatments, the most frequent adverse events were asthma worsening, nasopharyngitis, and bronchitis.

The aim of the BORA trial (ClinicalTrials.gov Identifier: NCT02258542) is to evaluate the safety and tolerability pattern of benralizumab in subjects with asthma already enrolled in either SIROCCO, CALIMA, or ZONDA.

The BISE study was carried out by Ferguson et al. in patients with mild-to-moderate persistent asthma, receiving low-to-medium ICS dosages, who were randomly treated via the subcutaneous route, every 4 weeks for 12 weeks, with either placebo or 30 mg of benralizumab [26]. With respect to placebo, at the 12th week, benralizumab induced a prebronchodilator FEV_1_ increase of 80 mL. FEV_1_ changes did not result to be different with regard to baseline blood eosinophil counts higher or lower than 300/*μ*L. Furthermore, differently from placebo, benralizumab completely and persistently (up to the 20th week) depleted blood eosinophils. Patient groups receiving benralizumab or placebo experienced similar incidences of adverse reactions, mostly consisting of upper respiratory tract infections, nasopharyngitis, headache, and asthma worsening.

The GREGALE open label trial has recently demonstrated that the majority of severe asthmatic patients and their family members, recruited in the study, were able at home to efficiently use an accessorized prefilled syringe, prepared for subcutaneous injections of benralizumab 30 mg [19].

A meta-analysis referring to 1951 subjects with eosinophilic asthma, enrolled in several different phase 1, 2, and 3 randomized controlled trials, demonstrated that, when compared to placebo, benralizumab induced significant score improvements of asthma control questionnaire-6 (ACQ-6) and asthma quality of life questionnaire (AQLQ) and enhanced FEV_1_ and also decreased the annual rate of disease exacerbations [72]. In addition, this meta-analysis showed that the dosage schedule of benralizumab 30 mg every 8 weeks produced better results than the same dose injected every 4 weeks. Furthermore, benralizumab and placebo induced similar patterns of adverse effects.

Besides the WINDWARD program, other ongoing phase 3 trials are ANDHI, MIRACLE, and SOLANA [19]. The ANDHI study (ClinicalTrials.gov Identifier: NCT03170271) aims to assess, in patients with severe eosinophilic asthma, the effects of benralizumab on asthma exacerbations, lung function, and quality of life. In addition, some subjects enrolled in this trial will be also studied with regard to the effects of benralizumab on relevant comorbidities of asthma, such as nasal polyposis and chronic rhinosinusitis. The MIRACLE study (ClinicalTrials.gov Identifier: NCT03186209) is another trial designed to primarily investigate, in severe asthmatics receiving medium-to-high doses of ICS/LABA medications, the eventual ability of benralizumab to lower the annualized rate of asthma exacerbations. SOLANA (ClinicalTrials.gov Identifier: NCT02869438) is currently assessing, in patients with severe eosinophilic asthma, the impact of benralizumab on symptom score, quality of life, lung function, and blood eosinophils.

Finally, a very recent study performed by Sehmi et al. on 18 patients with severe eosinophilic and corticosteroid-dependent asthma showed that 30 mg of benralizumab, administered subcutaneously every 4 or 8 weeks, when compared to placebo, significantly decreased the counts of mature eosinophils in both blood and induced sputum [73]. In blood, benralizumab also significantly reduced the number of eosinophil progenitors. A similar result was also detected in induced sputum, where, however, this effect of benralizumab did not reach the threshold of statistical significance, probably because of the small number of matched data sets [73]. Moreover, in blood, benralizumab significantly lowered the number of ILC2 cells expressing IL-5R*α*, and a similar effect was also observed in induced sputum, where, however, only a trend, and not a significant difference, was found. Serum EDN concentrations were also significantly diminished by benralizumab. In addition, benralizumab significantly increased the levels of granzyme B and interferon-*γ* in cell-free sputum supernatants. Therefore, the latter findings suggest that benralizumab was able to stimulate the activity of NK cells. All these biological effects of benralizumab were paralleled by relevant clinical and functional improvements, including a decrease in the maintenance dosage of oral corticosteroids, a better asthma control, and an increased ratio of prebronchodilator FEV_1_ to FVC (forced vital capacity).

## 5. Conclusions

It is currently clear that, given the pivotal functions exerted by IL-5 in the induction, maintenance, and amplification of airway eosinophilia driven by type-2 inflammation, such a cytokine and its receptor represent key molecules to be targeted by monoclonal antibodies with therapeutic properties of add-on biological treatments for severe eosinophilic asthma. In particular, because of its very effective action as IL-5R*α* antagonist, benralizumab has been shown to be capable of significantly inhibiting eosinophil differentiation in the bone marrow, as well as eosinophilic infiltration of airways. These eosinopenic effects are further potentiated by ADCC-mediated eosinophil apoptosis, operated by NK cells, and stimulated by benralizumab. At clinical and functional levels, such a dual mechanism of action of benralizumab translates into relevant improvements, including a significant decrease of asthma exacerbations, a better symptom control, a marked sparing effect on the intake of oral corticosteroids, and an important attenuation of airflow limitation [74–76]. All these features, associated with a very good safety and tolerability profile, make benralizumab a valuable therapeutic option for add-on biological treatment of severe eosinophilic asthma.

## Figures and Tables

**Table 1:** Benralizumab: summary of the main phase 3 clinical trials.

Authors and trial name	Duration	Number of patients	Main results
Bleecker et al. (2016) [23], SIROCCO	48 weeks	1205	Fewer asthma exacerbations, higher FEV_1_
FitzGerald et al. (2016) [24], CALIMA	56 weeks	1306	Fewer asthma exacerbations, higher FEV_1_
Nair et al. (2017) [25], ZONDA	28 weeks	220	Lower intake of oral corticosteroids, fewer asthma exacerbations
Ferguson et al. (2017) [26], BISE	12 weeks	211	Smaller numbers of blood eosinophils

## References

[1] Carr T. F., Zeki A. A., Kraft M. (2018). Eosinophilic and noneosinophilic asthma.

[2] Papi A., Brightling C., Pedersen S. E., Reddel H. K. (2018). Asthma.

[3] Pelaia G., Vatrella A., Busceti M. T. (2015). Cellular mechanisms underlying eosinophilic and neutrophilic airway inflammation in asthma.

[4] Holgate S. T., Wenzel S., Postma D. S., Weiss S. T., Renz H., Sly P. D. (2015). Asthma.

[5] Hasegawa K., Stoll S. J., Ahn J., Bittner J. C., Camargo C. A. (2015). Prevalence of eosinophilia in hospitalized patients with asthma exacerbation.

[6] De Groot J. C., Brinke A. T., Bel E. H. D. (2015). Management of the patient with eosinophilic asthma: A new era begins.

[7] Zhang X., Moilanen E., Kankaanranta H. (2000). Enhancement of human eosinophil apoptosis by fluticasone propionate, budesonide, and beclomethasone.

[8] Pelaia G., Vatrella A., Busceti M. T. (2016). Molecular and cellular mechanisms underlying the therapeutic effects of budesonide in asthma.

[9] Dunican E. M., Fahy J. V. (2017). Asthma and corticosteroids: time for a more precise approach to treatment.

[10] Pazdrak K., Moon Y., Straub C., Stafford S., Kurosky A. (2016). Eosinophil resistance to glucocorticoid-induced apoptosis is mediated by the transcription factor NFIL3.

[11] Molfino N. A., Gossage D., Kolbeck R., Parker J. M., Geba G. P. (2012). Molecular and clinical rationale for therapeutic targeting of interleukin-5 and its receptor.

[12] Varricchi G., Bagnasco D., Borriello F., Heffler E., Canonica G. W. (2016). Interleukin-5 pathway inhibition in the treatment of eosinophilic respiratory disorders: Evidence and unmet needs.

[13] Pelaia G., Vatrella A., Maselli R. (2012). The potential of biologics for the treatment of asthma.

[14] Gallelli L., Busceti M. T., Vatrella A., Maselli R., Pelaia G. (2013). Update on anti-cytokine treatment for asthma.

[15] Fulkerson P. C., Rothenberg M. E. (2013). Targeting eosinophils in allergy, inflammation and beyond.

[16] Bel E. H., ten Brinke A. (2017). New Anti-Eosinophil Drugs for Asthma and COPD: Targeting the Trait!.

[17] Pelaia C., Vatrella A., Busceti M. T. (2017). Severe eosinophilic asthma: From the pathogenic role of interleukin-5 to the therapeutic action of mepolizumab.

[18] Pelaia G., Vatrella A., Busceti M. T. (2016). Role of biologics in severe eosinophilic asthma – focus on reslizumab.

[19] Matera M. G., Calzetta L., Rinaldi B., Cazzola M. (2017). Pharmacokinetic/pharmacodynamic drug evaluation of benralizumab for the treatment of asthma.

[20] Shrimanker R., Pavord I. D. (2017). Interleukin-5 Inhibitors for Severe Asthma: Rationale and Future Outlook.

[21] Ray A., Raundhal M., Oriss T. B., Ray P., Wenzel S. E. (2016). Current concepts of severe asthma.

[22] Israel E., Reddel H. K. (2017). Severe and difficult-to-treat asthma in adults.

[23] Bleecker E. R., FitzGerald J. M., Chanez P. (2016). Efficacy and safety of benralizumab for patients with severe asthma uncontrolled with high-dosage inhaled corticosteroids and long-acting *β*2-agonists (SIROCCO): a randomised, multicentre, placebo-controlled phase 3 trial.

[24] FitzGerald J. M., Bleecker E. R., Nair P. (2016). Benralizumab, an anti-interleukin-5 receptor *α* monoclonal antibody, as add-on treatment for patients with severe, uncontrolled, eosinophilic asthma (CALIMA): a randomised, double-blind, placebo-controlled phase 3 trial.

[25] Nair P., Wenzel S., Rabe K. F. (2017). Oral glucocorticoid-sparing effect of benralizumab in severe asthma.

[26] Ferguson G. T., FitzGerald J. M., Bleecker E. R. (2017). Benralizumab for patients with mild to moderate persistent asthma (BISE): a randomised, double-blind, placebo-controlled phase 3 trial.

[27] Yanagibashi T., Satoh M., Nagai Y., Koike M., Takatsu K. (2017). Allergic diseases: From bench to clinic - Contribution of the discovery of interleukin-5.

[28] Woodruff P. G., Modrek B., Choy D. F. (2009). T-helper type 2-driven inflammation defines major subphenotypes of asthma.

[29] Brusselle G. G., Maes T., Bracke K. R. (2013). Eosinophilic airway inflammation in nonallergic asthma.

[30] Walker J. A., Barlow J. L., McKenzie A. N. J. (2013). Innate lymphoid cells-how did we miss them?.

[31] Smith S. G., Chen R., Kjarsgaard M. (2016). Increased numbers of activated group 2 innate lymphoid cells in the airways of patients with severe asthma and persistent airway eosinophilia.

[32] Lambrecht B. N., Hammad H. (2014). The immunology of asthma.

[33] Wood L. J., Sehmi R., Dorman S. (2002). Allergen-induced increases in bone marrow T lymphocytes and interleukin-5 expression in subjects with asthma.

[34] Dorman S. C., Efthimiadis A., Babirad I. (2004). Sputum CD34^+^IL-5R*α*^+^ cells increase after allergen evidence for in situ eosinophilopoiesis.

[35] Johansson M. W. (2017). Eosinophil Activation Status in Separate Compartments and Association with Asthma.

[36] Takatsu K., Tominaga A., Harada N. (1988). T Cell‐Replacing Factor (TRF)/Interleukin 5 (IL‐5): Molecular and Functional Properties.

[37] Milburn M. V., Hassell A. M., Lambert M. H. (1993). A novel dimer configuration revealed by the crystal structure at 2.4 Å resolution of human interleukin-5.

[38] Takatsu K., Takaki S., Hitoshid Y. (1994). Interleukin-5 and Its Receptor System: Implications in the Immune System and Inflammation.

[39] Rossjohn J., McKinstry W. J., Woodcock J. M. (2000). Structure of the activation domain of the GM-CSF/IL-3/IL-5 receptor common *β*-chain bound to an antagonist.

[40] Murphy J. M., Young I. G. (2006). IL-3, IL-5, and GM-CSF Signaling: Crystal Structure of the Human Beta-Common Receptor.

[41] Pazdrak K., Stafford S., Alam R. (1995). The activation of the Jak-STAT 1 signaling pathway by IL-5 in eosinophils.

[42] Stout B. A., Bates M. E., Liu L. Y., Farrington N. N., Bertics P. J. (2004). IL-5 and granulocyte-macrophage colony-stimulating factor activate STAT3 and STAT5 and promote Pim-1 and cyclin D3 protein expression in human eosinophils.

[43] Pazdrak K., Olszewska-Pazdrak B., Stafford S., Garofalo R. P., Alam R. (1998). Lyn, Jak2, and Raf-1 kinases are critical for the antiapoptotic effect of interleukin 5, whereas only Raf-1 kinase is essential for eosinophil activation and degranulation.

[44] Pazdrak K., Schreiber D., Forsythe P., Justement L., Alam R. (1995). The intracellular signal transduction mechanism of interleukin 5 in eosinophils: The involvement of lyn tyrosine kinase and the ras-raf-1-MEK-microtubule-associated protein kinase pathway.

[45] Adachi T., Alam R. (1998). The mechanism of IL-5 signal transduction.

[46] Takatsu K., Nakajima H. (2008). IL-5 and eosinophilia.

[47] Bates M. E., Green V. L., Bertics P. J. (2000). ERK1 and ERK2 activation by chemotactic factors in human eosinophils is interleukin 5-dependent and contributes to leukotriene C4 biosynthesis.

[48] Pelaia G., Cuda G., Vatrella A. (2005). Mitogen-activated protein kinases and asthma.

[49] Adachi T., Choudhury B. K., Stafford S., Sur S., Alam R. (2000). The differential role of extracellular signal-regulated kinases and p38 mitogen-activated protein kinase in eosinophil functions.

[50] Sano M., Leff A. R., Myou S. (2005). Regulation of interleukin-5-induced *β*2-integrin adhesion of human eosinophils by phosphoinositide 3-kinase.

[51] Bousquet J., Chanez P., Lacoste J. Y. (1990). Eosinophilic inflammation in asthma.

[52] Howarth P. H., Bradding P., Montefort S. (1994). Mucosal inflammation and asthma.

[53] Gleich G. J. (2000). Mechanisms of eosinophil-associated inflammation.

[54] McBrien C. N., Menzies-Gow A. (2017). The biology of eosinophils and their role in asthma.

[55] Koike M., Nakamura K., Furuya A. (2009). Establishment of humanized anti-interleukin-5 receptor alpha chain monoclonal antibodies having a potent neutralizing activity.

[56] Ghazi A., Trikha A., Calhoun W. J. (2012). Benralizumab - A humanized mAb to IL-5R*α* with enhanced antibody-dependent cell-mediated cytotoxicity - A novel approach for the treatment of asthma.

[57] Menzella F., Lusuardi M., Galeone C., Facciolongo N., Zucchi L. (2016). The clinical profile of benralizumab in the management of severe eosinophilic asthma.

[58] Ishino T., Pasut G., Scibek J., Chaiken I. (2004). Kinetic Interaction Analysis of Human Interleukin 5 Receptor *α* Mutants Reveals a Unique Binding Topology and Charge Distribution for Cytokine Recognition.

[59] Kolbeck R., Kozhich A., Koike M. (2010). Medi-563, a humanized anti-IL-5 receptor a mAb with enhanced antibody-dependent cell mediated cytotoxicity function.

[60] Shields R. L., Lai J., Keck R. (2002). Lack of fucose on human IgG1 N-linked oligosaccharide improves binding to human Fc*γ*RIII and antibody-dependent cellular toxicity.

[61] Shinkawa T., Nakamura K., Yamane N. (2003). The absence of fucose but not the presence of galactose or bisecting N-acetylglucosamine of human IgG1 complex-type oligosaccharides shows the critical role of enhancing antibody-dependent cellular cytotoxicity.

[62] Busse W. W., Katial R., Gossage D. (2010). Safety profile, pharmacokinetics, and biologic activity of MEDI-563, an anti-IL-5 receptor *α* antibody, in a phase I study of subjects with mild asthma.

[63] Laviolette M., Gossage D. L., Gauvreau G. (2013). Effects of benralizumab on airway eosinophils in asthmatic patients with sputum eosinophilia.

[64] Assa'ad A. H., Rothenberg M. E. (2013). Eosinophilic asthma: Insights into the effects of reducing IL-5 receptor-positive cell levels.

[65] Korosec P., Gibbs B. F., Rijavec M. (2018). Important and specific role for basophils in acute allergic asthma.

[66] Pelaia C., Vatrella A., Lombardo N. (2018). Biological mechanisms underlying the clinical effects of allergen-specific immunotherapy in asthmatic children.

[67] Park H.-S., Kim M.-K., Imai N. (2016). A phase 2a study of benralizumab for patients with eosinophilic asthma in South Korea and Japan.

[68] Castro M., Wenzel S. E., Bleecker E. R. (2014). Benralizumab, an anti-interleukin 5 receptor *α* monoclonal antibody, versus placebo for uncontrolled eosinophilic asthma: a phase 2b randomised dose-ranging study.

[69] Nowak R. M., Parker J. M., Silverman R. A. (2015). A randomized trial of benralizumab, an antiinterleukin 5 receptor *α* monoclonal antibody, after acute asthma.

[70] Pham T.-H., Damera G., Newbold P., Ranade K. (2016). Reductions in eosinophil biomarkers by benralizumab in patients with asthma.

[71] Chipps B. E., Newbold P., Hirsch I., Trudo F., Goldman M. (2018). Benralizumab efficacy by atopy status and serum immunoglobulin E for patients with severe, uncontrolled asthma.

[72] Liu T., Wang F., Wang G., Mao H. (2017). Efficacy and safety of benralizumab in patients with eosinophilic asthma: a meta-analysis of randomized placebo-controlled trials.

[73] Sehmi R., Lim H. F., Mukherjee M. (2018). Benralizumab attenuates airway eosinophilia in prednisone-dependent asthma.

[74] Kupczyk M., Kuna P. (2018). Benralizumab: an anti-IL-5 receptor *α* monoclonal antibody in the treatment of asthma.

[75] Tan L. D., Bratt J. M., Godor D., Louie S., Kenyon N. J. (2016). Benralizumab: A unique IL-5 inhibitor for severe asthma.

[76] Khorasanizadeh M., Eskian M., Assa’ad A. H., Camargo C. A., Rezaei N. (2016). Efficacy and Safety of Benralizumab, a Monoclonal Antibody against IL-5R*α*, in Uncontrolled Eosinophilic Asthma.

